# 3D imaging and anisotropy mapping of the lamb disc for biomechanical and regenerative insights

**DOI:** 10.3389/fphys.2026.1786152

**Published:** 2026-03-05

**Authors:** Ana Prates Soares, Andreia Sousa da Silveira, Jussi-Petteri Suuronen, Paul Helmerking, Timm Weitkamp, Bernhard Hesse, Katharina Schmidt-Bleek, Carsten Rendenbach

**Affiliations:** 1 Julius Wolff Institute for Biomechanics and Musculoskeletal Regeneration, Berlin Institute of Health at Charité - Universitätsmedizin Berlin, Berlin, Germany; 2 Department of Oral and Maxillofacial Surgery, Charité – Universitätsmedizin Berlin, Corporate Member of Freie Universität Berlin and Humboldt-Universität zu Berlin, Berlin, Germany; 3 Department for Operative, Preventive and Pediatric Dentistry – Universitätsmedizin Berlin, Corporate Member of Freie Universität Berlin and Humboldt-Universität zu Berlin, Berlin, Germany; 4 Xploraytion GmbH, Berlin, Germany; 5 Synchrotron SOLEIL, Saint-Aubin, France; 6 Berlin Institute of Health Centre for Regenerative Therapies (BCRT), Berlin Institute of Health at Charité - Universitätsmedizin Berlin, Berlin, Germany

**Keywords:** anisotropy, biomechanics, fibrocartilage, microtomography, synchrotron radiation, temporomandibular joint (TMJ)

## Abstract

**Background:**

The temporomandibular joint (TMJ) relies on a fibrocartilaginous disc for stabilization and load distribution. When the disc degenerates, current replacement options fail to restore native biomechanics. Developing effective implants requires detailed knowledge of the disc’s structure. The present work provides a full-volume, three-dimensional characterization of collagen fiber architecture and anisotropy in a large animal model with anatomical and functional similarities to the human joint.

**Methods:**

A multimodal 3D imaging workflow was implemented, combining cone-beam CT for anatomical context and synchrotron phase-contrast micro-CT for high-resolution visualization of the ovine temporomandibular joint disc, cartilage, ligament, and subchondral bone. Deep-learning segmentation enabled full-volume tissue segmentation. Fiber orientation and anisotropy were quantified using mean intercept length (Mean Intercept Length)–derived eigenvector fields, with analysis performed across anatomical axes and planes. Histological sections validated fiber segmentation and regional differences in extracellular matrix organization.

**Results:**

The lamb TMJ disc displayed a heterogeneous but highly ordered collagen network. Strong lateromedial alignment formed frontal-plane reinforcement bands, while a craniocaudal tensile corridor dominated the sagittal plane, and mixed lateromedial–ventrodorsal orientations characterized the transverse plane. Anisotropy was highest in the peripheral rims and lower in the central zone, reflecting a functional division between stabilization and deformation. Quantitative analysis demonstrated an orthotropic organization, with distinct dominant fiber populations aligned along the lateral–medial, ventral–dorsal, and cranial–caudal axes. Subchondral bone beneath the disc exhibited a fine, highly anisotropic trabecular lattice with reduced spacing, complementing the disc’s structural organization.

**Conclusion:**

This study provides the first full-volume, plane-resolved 3D description of collagen anisotropy in the ovine TMJ disc. The orthotropic fiber architecture and regional anisotropy gradients identified here clarify direction-dependent mechanical behavior and offer quantitative benchmarks for the design of biomimetic scaffolds and regenerative TMJ disc replacements.

## 1 Introduction

The temporomandibular joint (TMJ) is a complex synovial joint that enables chewing, speech, and various intricate jaw movements. It consists of the condylar process of the mandible and the glenoid fossa of the temporal bone, connected by ligaments and interposed by a fibrocartilaginous disc (Patil and Bindra, 2012). This disc helps absorb and distribute mechanical loads during joint activity, deforming to support movements in multiple directions (Tanaka and Van Eijden, 2003). Degenerative TMJ disorders often involve progressive deterioration of the disc, leading to a loss of its natural mechanical integrity (Cui et al., 2025). Disc deterioration is characterized by the breakdown of collagen and elastin networks within the disc, resulting in significant reductions in tensile stiffness and strength, as well as altered ultrastructure (Cui et al., 2025). While conservative and surgical treatments can partially restore joint function, neither approach fully recovers the disc’s original dynamics (De Riu et al., 2013). Symptomatic relief through conservative therapy or disc surgery does not restore the native anisotropic structure or mechanical behavior of the disc (Singh et al., 2022). Therefore, developing regenerative strategies that can replicate the disc’s anisotropic architecture and load-bearing capabilities has become a significant research goal (She et al., 2024).

Large-animal models, such as sheep, serve as a crucial platform for studying TMJ biomechanics and testing tissue-engineered scaffolds because their joint size, fibrocartilaginous disc shape, and overall structure resemble those of humans (Socorro et al., 2025). The ovine TMJ disc has a biconcave shape with thicker anterior and posterior bands and a thinner intermediate zone, similar to the human disc (Patil and Bindra, 2012). Histologically, both species exhibit a fibrocartilaginous composition, mainly composed of type I collagen, with fiber orientation that varies regionally, allowing for anisotropic mechanical responses to complex mandibular loads (Tanaka and Van Eijden, 2003). However, notable morphological and functional differences show adaptations to ruminant chewing. In sheep, the mandibular condyle has a mediolateral concavity, unlike the convex shape found in humans, and the articular eminence is underdeveloped (Patil and Bindra, 2012). Functionally, the ovine TMJ is optimized for large lateral movements and rotation within the transverse plane, with limited vertical opening, ideal for prolonged chewing and rumination cycles that total around 4 h of mastication and 8 h of rumination daily (Herring, 2003). Despite these functional differences, comparative studies show that the ovine TMJ mimics human-like mechanical characteristics and fibrocartilaginous organization, supporting its use as a preclinical model for TMJ biomechanics and disc tissue engineering (Angelo et al., 2016).

Recent advances in imaging and computational modeling have enabled detailed three-dimensional reconstructions of the temporomandibular joint (TMJ) and its disc (Savignat et al., 2025), enhancing understanding of joint morphology and movement. Magnetic resonance–based methods have been used to visualize the spatial relationships among the condyle, glenoid fossa, and disc in 3D, providing insights into disc position and deformation during jaw movement (Chirani et al., 2004; Costa et al., 2008). Dynamic and real-time MRI have enabled functional visualization of TMJ motion beyond static imaging, and recent studies demonstrate that modern low-field systems (e.g., 0.55 T) can provide diagnostically usable TMJ images (Vogl et al., 2021; Nixdorf et al., 2025). At the microscale, efforts to examine the collagen network within the disc have used diffusion tensor imaging to determine fiber orientation (Benavides et al., 2009), magnetic resonance–histology correlation to evaluate regional anisotropy (Eder et al., 2018), and second-harmonic generation microscopy to observe three-dimensional collagen fiber remodeling in degenerated fibrocartilage (Reed et al., 2019). While these methods have advanced understanding of local fiber organization, they are still limited to partial regions or two-dimensional views of inherently three-dimensional structures.

A comprehensive, whole-volume quantification of the TMJ disc’s three-dimensional collagen anisotropy has not yet been accomplished—especially in large-animal models like sheep, which are vital for translational research in TMJ biomechanics and tissue engineering. Filling this knowledge gap is crucial for linking structural anisotropy to joint mechanics and for guiding the development of biomimetic scaffolds capable of restoring the disc’s native load-bearing function. Therefore, this study presents a full-volume, three-dimensional characterization of collagen fiber architecture and anisotropy in the ovine temporomandibular joint (TMJ) disc, using high-resolution imaging and computational analysis to clarify its structural physiology and biomechanical relevance for disc degeneration and reconstruction. We hypothesize that the ovine TMJ disc exhibits a complex, region-dependent three-dimensional collagen anisotropy that can be resolved through comprehensive volumetric imaging and analysis.

## 2 Materials and methods

### 2.1 Sample preparation

A total of four lamb heads (aged approximately 6–8 months) were collected from a licensed butcher following standard slaughter procedures for human consumption; thus, no animals were explicitly sacrificed for this research. All samples had their jaw and TMJ dissected to assess the standardization of anatomical dimensions.

### 2.2 3D imaging acquisition

**FIGURE 1:**
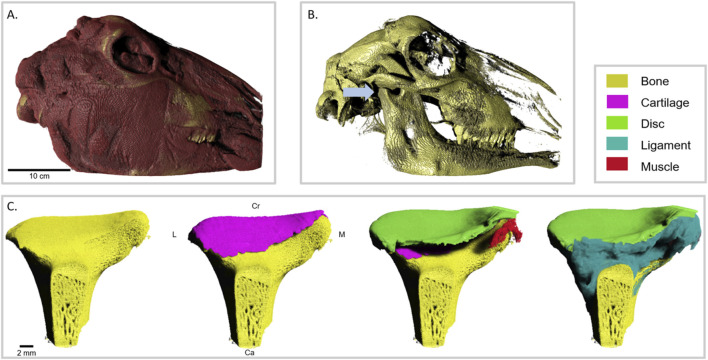
Three-dimensional rendering of the lamb TMJ: **(A)** Volume rendering of the whole head showing external soft-tissue contours. **(B)** Segmented cranial skeleton containing the right TMJ (blue arrow). **(C)** Sequential visualization of the mandibular condyle complex showing the segmented bone and associated soft-tissue structures extracted from the same dataset. From left to right: bone only; bone with articular cartilage; addition of the articular disk and the lateral pterygoid muscle attachment; and inclusion of the ligamentous tissue. Color coding: bone (yellow), articular cartilage (magenta), disk (green), ligament (cyan), muscle (red). Directional axes are labeled on the second redering of C (L, lateral; M, medial; Cr, cranial; Ca, caudal).

Cone Beam Computed Tomography (CBCT, Axeos, Dentsply Siron, at 89 µm pixel size) was performed on a selected lamb head to map the relationship between the joint, cranium, and face of the animal (Figures 1A,B). After examining the images, the right TMJ, condyle, disc, and connecting ligament were carefully harvested for further imaging. Sample preparation was performed by a single experienced operator.

To reveal the geometry of the condyle head and TMJ disc and how they are connected, phase-enhanced contrast micro-CT was performed at Synchrotron SOLEIL (Saint-Aubin, Essonne, France), the French national synchrotron radiation facility. The experiment was conducted at the ANATOMIX beamline (Weitkamp et al., 2022) using a filtered white beam with an average photon energy of approximately 45 keV. Each sample was imaged in a tomographic scan consisting of 4,500 projections evenly distributed over 360°. The large number of projections accounted for the offset between the rotation axis and the detector center, enabling an extended field of view through the “half-acquisition” approach. The exposure time for each projection radiograph was 100 m. Radiographs were recorded using a Hamamatsu Orca Flash 4.0 V2 camera (2048 × 2048 pixels) coupled to a 600-µm-thick lutetium aluminum garnet (LuAG, Lu_3_Al_5_O_12_, supplier: Crytur, Turnov, Czech Republic) scintillator via a 1:1 visible-light optics system, resulting in an isotropic voxel size of 6.48 µm. The sample-to-scintillator distance was approximately 1 m. Tomographic reconstruction was performed using the standard ANATOMIX data processing pipeline, which includes in-house software for pre-processing and the PyHST2 program (version 2021c, ESRF, Grenoble, France (Mirone et al., 2014)) as the backend for reconstruction. Reconstruction was carried out via the filtered back-projection algorithm, following phase retrieval using Paganin’s single-distance method (Paganin et al., 2002). The “Paganin length” parameter in PyHST2 was set to 40 pixels. After the phase retrieval step, an unsharp mask was applied with a Gaussian kernel (σ = 3 pixels) and a weight = 0.65 (Mölich et al., 2025). Image acquisition at the synchrotron required the work of a beamline scientist and two experimental researchers across two experiments to determine the optimal acquisition settings.

### 2.3 3D analysis pipeline

The 3D volumes were analyzed using Dragonfly Research (Version, 2024.1; Object Research Systems, Montreal, Canada). Segmentation was performed using the built-in deep learning (DL) segmentation tool of *Dragonfly Research*, which trained a U-Net convolutional neural network (CNN) model for automated tissue classification and region delineation into four major parts: condyle bone, TMJ disc, cartilage, and the discal ligament (marked in Figure 1C). Fiber bundles of the segmented TMJ disc were further segmented with another trained model using the DL segmentation tool *to classify all fibers.* Automated results were inspected, and, when needed, minor manual edits were made to ensure segmentation accuracy. Two experienced researchers participated in 3D segmentation and analysis, including validation of the segmentation.

### 2.4 Fiber orientation quantification

Fiber architecture was evaluated using the Bone Analysis module of Dragonfly by computing the Mean Intercept Length (MIL) from vector fields to map principal orientation, and deriving anisotropy magnitude from the corresponding outputs as a fabric tensor. The Bone Analysis workflow implements and applies anisotropy-mapping methods derived from classic stereology and trabecular-bone microarchitecture. Anisotropy is estimated by sampling the average distance between material–void interfaces along many directions; principal directions and degree of anisotropy are taken from the resulting fabric tensor. The settings followed the manufacturer’s guidance for MIL sampling and vector-field visualization, as well as their publication on the topic (Reznikov et al., 2022). The results were exported as a CSV file containing eigenvector components and associated eigenvalues and were further analyzed using a custom Python pipeline to implement comprehensive three-dimensional characterization and visualization.

Vector magnitudes and unit directional vectors were computed for each fiber represented by the eigenvectors. An anisotropy index was measured on a quantitative scale from 0 (isotropic) to 1 (highly anisotropic). For each point, the first principal component (primary eigenvector) of the MIL fabric tensor was extracted to represent the dominant fiber orientation. The vector magnitude was derived from the primary eigenvalue, representing the structural coherence and intensity of fiber alignment along this principal axis. The anisotropy index was calculated as a normalized ratio of the eigenvalues (scale 0–1), describing the degree of directional dominance relative to the transverse axes. Thus, while magnitude reflects the strength of the alignment, anisotropy reflects the exclusivity of that direction. Fiber principal component directions and magnitudes were characterized through: (1) primary anatomical axes (X: lateral-medial; Y: ventral-dorsal; Z: cranial-caudal) and (2) projection onto three anatomical planes (sagittal YZ, frontal XZ, and transverse XY). The analysis generated comprehensive visualization plots to identify patterns of dominant orientations and their associated magnitudes and anisotropies, including frequency histograms of directional angles for each anatomical plane and overall anisotropies, orientation distribution plots of each anatomical axis and anatomical plane. Additionally, directional distributions were visualized using rose diagrams to capture two distinct structural properties within each anatomical place: (1) Fiber Frequency, representing the structural geometry (fiber count in each direction), and (2) Sum Magnitude, calculated as the sum of vector magnitudes for all fibers in a given direction. While frequency indicates where fibers are located, sum magnitude serves as a proxy for the total reinforcement capacity of the tissue along that trajectory, accounting for both fiber density and the strength of alignment. Two researchers participated in the development and implementation of the 3D analysis pipeline.

### 2.5 Histological processing

After 3D imaging, the sample was decalcified in an EDTA solution (Carl Roth GmbH & Co. KG, Karlsruhe, Germany) for 7 weeks at 37 °C. It was then dehydrated through an ascending alcohol series and infiltrated with paraffin using a tissue processor (Leica TP 1020, Leica Biosystems GmbH, Nussloch). Following embedding, 4 μm-thick histological sections were cut with a microtome (Leica Biosystems Nussloch GmbH, Nussloch, Germany) along the frontal and sagittal planes. The samples were then stained with hematoxylin and eosin (H&E). Micrographs were captured with a digital light microscope (Leica DM6B, Leica Microsystems CMS, Wetzlar, Germany) connected to a digital camera (Leica DMC 4500, Leica Microsystems (Switzerland) Ltd., Heerbrugg, Switzerland), using the LASX software (Leica Application Suite X, Version: 3.7.5.24914). Systematic images were captured at ×100 magnification and seamlessly combined utilizing the system’s automatic mosaic stitching. For the histological processing and analysis, a single experienced operator led the work.

### 2.6 Statistical analysis

Statistics analysis included total vector count, percentage distributions of dominant directional components, mean and median anisotropy indices, and standard deviations.

## 3 Results

### 3.1 3D anatomic overview

The assembled data revealed the overall morphology of the hard- and soft-tissue architecture of the lamb TMJ, with particular focus on the geometry of the articular disc and the heterogeneous organization of its fiber orientations.

The lamb’s head CBCT volume highlighted the bone structure and some of the associated muscle tissue. A mostly symmetrical bone structure of both TMJs was observed (Figures 1A,B). After selecting the right TMJ and performing excision, the joint was imaged using phase-contrast micro-CT. The resulting volume was used to segment the tissues that make up the TMJ: bone, cartilage, ligament, muscle insertion, and disc (Figure 1C).

Following segmentation, the different tissues were evaluated for their morphology. The trabecular condyle bone was characterized by a mean anisotropy of 0.85, a volume of 584 mm^3^, a maximum width of 20.76 mm, and a height of 15.68 mm. The trabecular spacing and thickness were uneven, with smaller gaps and thinner trabeculae (minimum thickness of 17 µm) in areas contacting cartilage. Thicker trabecular (a maximum of 367 µm) and larger spacing were observed at the neck area of the condyle. The surface topographies of bone and cartilage were closely aligned along a continuous boundary, while the ligament contacted a more porous region of the bone surface.

The volumetric imaging demonstrated that the cartilage had a total volume of 87.39 mm^3^ and a maximum thickness of 0.68 mm. It covered the surface of the condylar head, interposing between the disc and the bone. The ligament interconnects the disc with cartilage and bone. On the volumes, the medial area of the ligament enclosed the lateral pterygoid muscle insertion to the disc (Figure 1C, red detail on the third image from the left). The disc had a width of 21.72 mm, a depth of 12.78 mm, and a maximum thickness of 1.63 mm, with a total volume of 278 mm^3^.

**FIGURE 2:**
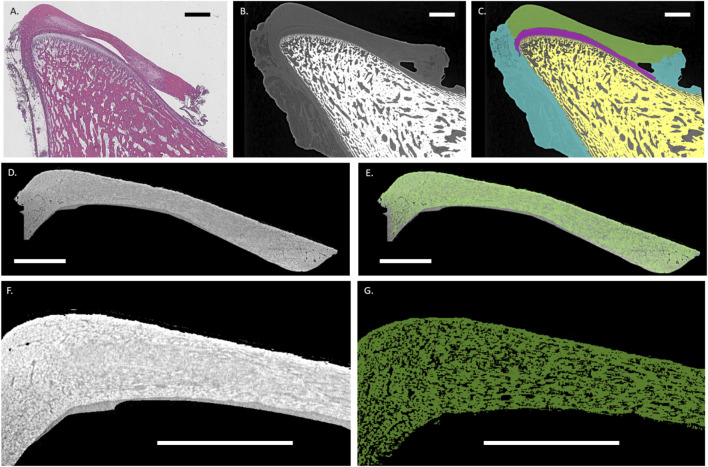
Histological and virtual slices of the TMJ condyle and associated tissues highlighting the condylar disc fibers: **(A)** Representative histological sagittal section (H&E stain) of the mandibular condyle and associated joint tissues illustrating the articular cartilage, disc, and adjacent bone. **(B)** Corresponding micro-CT slice. **(C)** Segmentation overlay of micro-CT data showing anatomical boundaries of the condylar bone (yellow), articular cartilage (magenta), articular disc (green), and ligamentous tissue (cyan). **(D)** Micro-CT sagittal slice of the segmented articular disc. **(E)** Overlayed segmented fibers (green) from the articular disc. **(F)** Highlight of the posterior band of the disc showing the fiber distribution. **(G)** Corresponding segmented fibers (green). Scale bar = 2 mm.

Fiber analysis of the TMJ disc revealed its complex, woven structure. When juxtaposed with the histological micrographs, the segmentation could be validated (Figures 2A–C; Supplementary Figure S1). Although the cellular component of the tissue was not visible in the 3D images, the fiber bundles were clearly seen. The fibers in the histological slices showed a wavier structure, likely due to tissue processing (Turunen et al., 2017). Additionally, during histological processing and sample cutting, the ligament lost its insertion, leading to reduced tissue tension and possibly further increasing collagen waviness. Areas of lower fiber density in the 3D volumes were observed in histological slices as more cellularized regions with a looser extracellular matrix, specifically located on the anterior and posterior bands of the disc (Figures 2D–G). Anatomically, while the anterior band lies under the articular eminence, the posterior band faces the temporal fossa. Because the fiber bundles were interconnected in a complex structure, segmenting individual collagen fibers throughout the entire disc was not feasible. Therefore, an eigenvector field using MIL was used to define the dominant bundle direction and degree of alignment and dispersion.

**FIGURE 3:**
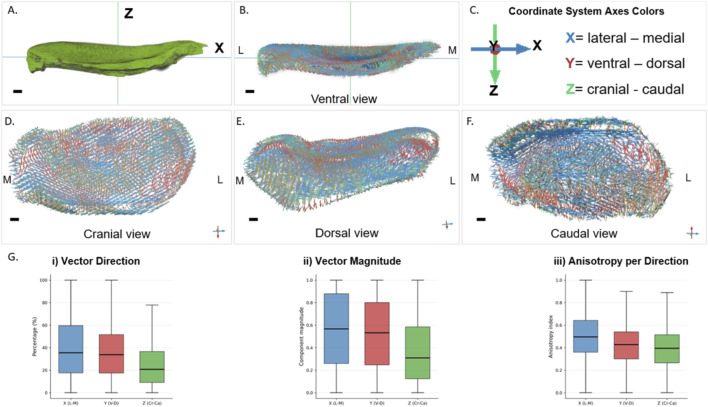
Fiber orientation mapping and quantitative directional analysis of the TMJ disc: **(A)** Volume rendering of the TMJ disc highlighting the cranial-caudal (Z-axis), and lateral-medial (X-axis) orientations. **(B)** Corresponding 3D renderings of the disc orientation vectors from a ventral view. **(C)** Reference coordinate system showing axis direction and color coding: X = lateral–medial (blue), Y = ventral–dorsal (red), Z = cranial–caudal (green). **(D–F)** Cranial, dorsal, and caudal perspective views revealing regional variations in fiber orientation and curvature. Fiber vectors are color-mapped to their dominant anatomical axis component, and intermediate directions have blended colors. **(G)** Quantitative analysis of disc fiber orientation. Left to right: (i) vector direction frequency demonstrating proportion of vectors oriented along each anatomical axis; (ii) magnitude-weighted component distribution for vectors aligned with each axis; (iii) anisotropy index per directional component. Scale bar = 1 cm.

The vector mapping of the disc fibers revealed a highly organized yet regionally heterogeneous structural arrangement of the fibers across the tissue (Figures 3A–F). When comparing lateral and medial areas, the vector field differed in direction and curvature, showing that fiber organization is not symmetrical. In the medial area, the fibers rotated, showing greater curvature. They converge, extending toward the disc’s medial boundary. This pattern reflects the anatomical continuity with the fibers of the neighboring muscular insertion. On the lateral area, fibers gradually curve along the external margin, extending toward the disc boundary and slightly wrapping the edge, demonstrating a coherent peripheral flow. The ventral side of the disc mainly has fibers oriented in a cranio-caudal direction along the thickness of the disc. The shift in fiber orientation toward the cranial and caudal ends happens gradually, with no sudden changes. On the dorsal side, the fibers follow the surface shape, bending laterally near the edges and showing a wider angle spread, smoothly transitioning into the cranial part of the disc. On the caudal side of the disc, the fibers display a strong medio-lateral component that decreases as they approach the margins, reflecting the shape of the caudal surface. The fibers contour along the caudal edge with smooth curves that follow the margin and blend into the lateral and medial surfaces. The cranial part of the disc showed more pronounced curvature, especially on the posterior band of the disc. Fibers curve outward from the central area toward both ventral and dorsal margins, following the surface’s concave–convex shape, with gradual rather than sudden changes in direction. In the anterior band of the disc, the fibers are more oriented laterally-medially. On the posterior band, which is the most convex part of the disc, the fibers have a more ventro-dorsal curvature, transitioning seamlessly into the caudal surface. The broad shape of the fiber direction forms a structural contour with localized adaptations that allow the disc to conform to TMJ movements while stabilizing it in place (Supplementary Figure S2, middle column). Further analysis and anisotropy mapping confirm that while the peripheral rims are the most anisotropic, functioning as reinforcement rings that stabilize the disc under complex, cyclic masticatory stresses, the central regions are more isotropic, accommodating deformation and acting as energy-dissipating zones (Supplementary Figure S2, right column).

Further analysis of the eigenvector field was performed to quantify the fiber content of the disc (Figure 3). The distribution of fiber directional components into three different axes (X, Y, and Z) revealed that the lateromedial (L–M; X-axis) alignment accounted for the highest percentage of the overall fiber orientation, followed by the ventral–dorsal (V–D; Y-axis) direction, while the craniocaudal (Cr–Ca; Z-axis) direction accounted for the least. This indicates that when orientation is resolved into anatomical axes, the L–M direction is the most frequently dominant component (Figures 3G–i). When examining the sum magnitudes of the directional components representing the strength or confidence of fiber alignment in 3D space, the L–M direction again showed the highest magnitudes, with V–D showing moderately high magnitudes, and Cr–Ca consistently lower magnitudes. Thus, when fibers are aligned in L–M or V–D directions, their orientation is also more strongly and coherently defined. In contrast, Cr-Ca oriented fibers tend to be weaker or less uniformly oriented (Figures 3G-ii). The anisotropy index supported this pattern by showing that the L–M direction possessed the most structurally ordered fiber organization. Although anisotropy was present along all axes, the V–D and Cr–Ca planes components displayed lower median anisotropy and wider variability, indicating less uniform structural alignment along those axes compared to the more consistently ordered L–M architecture (Figures 3G–iii). The distribution of directional components (Figures 3G–i) and their associated magnitudes (Figures 3G–ii) confirm an orthotropic organization of the collagen network. Together, these results support an orthotropic organization of the collagen network, in which each anatomical axis contributes a distinct, unlinked directional role (Figure 3). This indicates that the collagen fibers are not preferentially aligned along any combined axis but instead display a complex, multidirectional architecture. While most fibers align predominantly along the X-axis, their collective arrangement deviates from simple anisotropy, reflecting an orthotropic rather than uniaxially anisotropic structure (Supplementary Video S1).

**FIGURE 4:**
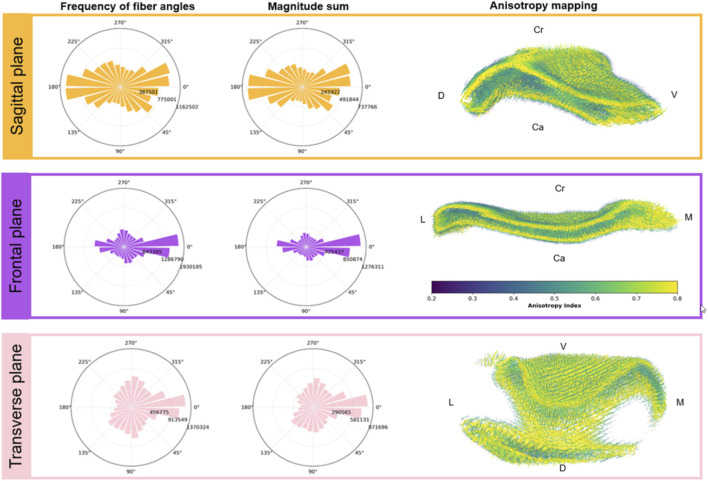
Fiber orientation distributions and anisotropy mapping of the articular disc across anatomical planes: Fiber orientations were quantified in three orthogonal anatomical planes: sagittal (top row), frontal (middle row), and transverse (bottom row). Left column: circular histograms showing the frequency of fiber angles within each plane. The middle column shows the sum magnitude distributions representing the total alignment strength (sum of vector magnitudes) in each direction. The strong resemblance to the frequency plots (left column) suggests that directional reinforcement is dominated by fiber quantity (density) rather than regional variations in alignment quality. Right column: spatial anisotropy mapping overlaid on the surface geometry, color-coded by the anisotropy index (0.2–0.8), where higher values indicate increased directional alignment. Renderings have been cut to show the internal aspect of the anisotropy maps. Anatomical direction references are labeled: cranial (Cr), caudal (Ca), dorsal (D), ventral (V), lateral (L), and medial (M).

Analysis of fiber architecture across different anatomical planes enabled visualization of fiber directions and the correlation between TMJ morphology and biomechanics (Figure 4; Supplementary Figure S3). In the sagittal plane (Z and Y-axes, or Cr-Ca and V-D), the frequency distribution of fiber orientations (Figure 4, top panel) indicated a substantial prevalence of fibers aligned approximately along the ventrodorsal axis. When weighted by orientation magnitude, this dominant alignment becomes even more apparent, demonstrating that fibers oriented craniocaudally are not only common but also more confidently and coherently defined than in other directions in this plane. The anisotropy map reveals a peak along the central craniocaudal band, extending from the ventral to dorsal edges. This pattern defines the disc’s tensile axis. Above and below this yellow tract, anisotropy decreases to green and blue, showing that the superior and inferior surfaces are more compliant and less organized, an expected adaptation to compressive deformation and sliding contact with the condyle and fossa.

In the frontal plane (Z and X-axes, or Cr-Ca and L-M), the fiber orientation analysis revealed a dominant lateromedial orientation across the disc (Figure 4, middle panel). Similarly, the sum magnitude distribution displayed a profile nearly identical to the frequency plot. This concordance confirms that the lateromedial axis is reinforced by a massive recruitment of aligned fibers, rather than by a selective increase in individual fiber stiffness. The anisotropy map highlights a highly coherent lateromedial fiber band, with lower anisotropy in the peripheral and central domains. This indicates that the frontal plane fiber network is structurally organized to resist L-M shear forces and maintain joint stability during mediolateral grinding, which is the leading movement of ruminant mastication.

Finally, in the transverse plane (Y and X-axes, or V-D and L-M), fibers again show a prominent lateromedial component, though with greater multidirectional spread than in the frontal plane (Figure 4, bottom panel). The sum magnitude distribution retained the L–M preference and secondary dorsoventral contribution seen in the frequency plot, reflecting the combined shear and compression environment acting across the disc thickness. Anisotropy mapping shows that highly organized L-M fibers dominate the posterior and lateral rims, while increased directional variability appears centrally and ventrally. This mixed-direction organization suggests that the transverse-plane fibers contribute to both lateral force dissipation and vertical load distribution, protecting the disc against compressive deformation during occlusal loading (chewing).

## 4 Discussion

The present study provides the first full-volume, plane-resolved three-dimensional analysis of collagen fiber orientation and anisotropy in the ovine temporomandibular joint disc. The results describe a region-dependent collagen organization with graded anisotropy, providing quantitative structural data to contextualize disc biomechanics and guide preclinical scaffold development. The current findings broadly agree with previous reports. The dimensions and morphology of the lamb TMJ, especially the disc with its biconcave profile and central thinning, closely align with prior characterizations (Angelo et al., 2016). The 3D segmentation further confirms the classical disc attachments and the medial insertion of the lateral pterygoid, as described in ovine dissection studies (Angelo et al., 2016), while providing a volumetric representation of the ligament–muscle–disc assembly. Using synchrotron phase-contrast micro-CT allowed for detailed visualization of various tissues in the current sample, thanks to its high sensitivity.

The 3D micro-CT-based eigenvector analysis demonstrates that the lamb TMJ disc exhibits a highly organized yet regionally heterogeneous collagen architecture. At the microstructural level, the eigenvector-based fiber mapping reveals circumferentially oriented, highly anisotropic peripheral rims and a more isotropic central region. This pattern is consistent with prior histological reports (Lee et al., 2022) and with less fiber-dense anterior and posterior zones (Lee et al., 2022). These findings are consistent with human data from Gutman et al. (2018), who reported region-dependent collagen orientations, with stronger alignment in the anterior band and intermediate zone than in the posterior, medial, and lateral regions. However, by resolving fiber trajectories in three dimensions, the present data reveal that zones previously described as ‘poorly aligned’ in 2D actually contain strongly anisotropic fibers that reorient over short distances to follow articular surfaces and muscle/ligament insertions, especially medially. Furthermore, the present data extend these findings by showing that the collagen network is orthotropic: fibers predominantly align with the lateral–medial, ventral–dorsal, and cranial–caudal directions in different regions of the TMJ disc, suggesting that each anatomical axis fulfills a distinct, mechanically relevant role.

This orthotropic structure offers a basis for understanding the region and direction-specific mechanical properties reported in ovine TMJ discs (Angelo et al., 2016; Lee et al., 2022). In whole-disc uniaxial tests, Angelo et al. (2016) measured higher lateromedial tensile moduli than ventrodorsal moduli, consistent with ruminant laterotrusive loading. Labus et al. (2021), using central dog-bone specimens at full disc thickness, reported a higher ventrodorsal modulus relative to lateromedial, and greater outer band than disc center stiffness in indentation. The present whole-disk orientation fields show a central craniocaudal/anteroposterior tensile band embedded within lateromedial peripheral reinforcement, explaining how the region-of-interest selection and testing mode can invert the apparent principal direction while preserving the underlying orthotropy. Furthermore, the observed anisotropy gradient aligns with viscoelastic behavior observed by Labus et al.’s (2021) quasi-linear viscoelastic fits, with regions with lower alignment in the disc center displaying greater relaxation.

The ovine temporomandibular joint shares several structural features with the human joint, including a fibrocartilaginous disc, biconcave morphology, and region-dependent collagen organization (Patil and Bindra, 2012; Angelo et al., 2016; Gutman et al., 2018). However, differences in joint geometry and masticatory function, particularly the predominance of lateromedial motion in ruminants, limit direct quantitative comparison of absolute fiber orientations (Herring, 2003). For this reason, the ovine TMJ is best viewed as a preclinical model in which general organizational patterns of disc architecture can be examined and cautiously considered in the context of human TMJ biomechanics and reconstruction. Furthermore, while magnetic resonance imaging is the standard clinical modality for TMJ assessment in humans (Chirani et al., 2004; Costa et al., 2008), its spatial resolution and sensitivity to collagen fiber architecture remain limited compared to synchrotron-based phase-contrast micro-CT, which therefore serves an essential role at the preclinical structural level.

Anisotropy and collagen fiber directionality are key determinants of biomechanical behavior in fibrous tissues, governing how these materials respond to tensile, compressive, and shear loads (Abraham et al., 2011). The present findings show that cumulative magnitude distributions closely mirror fiber frequency (Figure 4), suggesting that the TMJ disc reinforces these dominant loading axes primarily through fiber recruitment, rather than by altering the individual alignment quality of the collagen bundles. In tendons and ligaments, regions with highly aligned collagen fibers show greater tensile stiffness and strength along the fiber axis compared to transverse directions (Thomopoulos et al., 2006; Lake et al., 2010), while in intervertebral discs, fibers actively reorient under tensile load to align with the direction of strain, optimizing stress distribution and mechanical efficiency (Guerin and Elliott, 2006). The current 3D experimental method for visualizing anisotropy through both magnitude and directional analysis is an advanced technique for fibrous tissue characterization. The vector field and anisotropy mapping capture the dynamic organizational matrix of the TMJ disc and enable linking tissue microstructure with mechanical function. Therefore, anisotropic analysis is crucial for understanding the mechanical behavior of the TMJ disc under physiological conditions. Research demonstrates that stress patterns in the TMJ disc change continuously during jaw movement through human dynamic finite element and jaw motion models (Rupp et al., 2010; Hallman et al., 2011; Halilaj et al., 2014; Cibis et al., 2015). The architecture of the fibers observed in the sagittal, frontal, and transverse planes allows identification of primary and secondary loading directions, which are essential for understanding how the disc accommodates complex mandibular movements during function. The frontal plane highlights the dominant load-bearing ring architecture, which is critical for distributing lateral translational forces between the condyle and the fossa during prolonged horizontal ruminating cycles. While the sagittal-plane fiber orientation supports tensile loading during mandibular opening and closing, condylar translation exerts forces along the ventrodorsal axis.

A further comparison of the current data with the spatial distribution of TMJ implant wear reported by De Meurechy et al. (2022) shows a close alignment between the anisotropic architecture and the loading areas identified in the previous study. In the current data, the organization of the eigenvector field indicates that during mastication, shear and compressive forces concentrate within the central corridor and are guided lateromedially across the disc thickness. Correspondingly, De Meurechy et al. (2022) observed a principal wear in the fossa and often oriented diagonally in the lateromedial direction, precisely mirroring the primary vector field in the frontal and transverse planes. An additional anterior wear line reported at the coronoid-contact region matches the curved fiber trajectories of the anterior band beneath the articular eminence, where edge loading naturally occurs during wide opening and protrusion. Likewise, the occasional posterior wear tracks in displaced fossae correspond to the caudal band of the disk, whose fibers exhibit a strong medio-lateral curvature that guides posterior translation. Thus, the wear patterns seen on the prosthetic joint replicate the internal force pathways encoded by the anisotropic collagen network of the native disk, validating that the same lateromedial and craniocaudal mechanical axes govern the dominant contact and shear forces in both native and prosthetic TMJs.

Finally, by considering the trabecular compartment together with the disc, the present dataset enables a broader mechanobiological interpretation. The subchondral region of the condylar head displayed a fine, highly anisotropic trabecular lattice with reduced spacing and thinner trabeculae directly beneath the cartilage–disc interface, a pattern previously shown to align with principal TMJ loading trajectories (Ben-Zvi et al., 2017). When viewed alongside the disc’s orthotropic organization described above, this directional ordering suggests that both tissues may be shaped by the same dominant loading environment rather than acting as mechanically isolated structures. This interpretation is consistent with reports that TMJ disc collagen architecture and mechanical behavior are strongly direction-dependent (Gutman et al., 2018; Labus et al., 2021). Although we did not quantify a direct correspondence between disc and trabecular fabric tensors, their anatomical co-localization is compatible with Wolff’s law and supports a testable hypothesis of coordinated adaptation within the disc–bone functional unit.

Attempts to exploit the natural arrangement of the disc to achieve near-native function in implants have used different approaches, like directionally biased loading to generate *in vitro* collagen-aligned scaffolds (MacBarb et al., 2013), 3D printing with region-variant filament orientation (Legemate et al., 2016), and decellularized disc matrix reinforced by polycaprolactone (Jiang et al., 2023). The assembled data support the clinical development of TMJ implants and scaffold designs that maximize rim strength, preserve central compliance at a given thickness, and maintain through-thickness organization in an orthotropic manner. The data provide design-level targets for future scaffolds (e.g., where and how firmly to align printed fibers, where to allow more isotropy, how to handle transitions around insertions), as well as a structural framework for interpreting multiaxial mechanical tests and *in vivo* loading. In this way, the present work helps relate idealized, directionally reinforced constructs to the three-dimensional orthotropic architecture observed in the native TMJ disc, providing structural context that may support the development and evaluation of engineered replacements in preclinical settings.

The present study is limited by the number of specimens analyzed with full-volume synchrotron imaging and should therefore be interpreted as a high-resolution structural characterization rather than a population-level assessment. In addition, although the ovine temporomandibular joint shares key structural features with the human joint, species-specific differences in joint geometry and loading constrain direct quantitative extrapolation, and the findings are best viewed in a preclinical context.

## 5 Conclusion

Together, the current data show that the TMJ disc does not act as a simple transversely isotropic membrane, but rather as a 3D orthotropic network in which peripheral reinforcement rings and central energy-dissipating zones are connected to bone via insertional anatomy. These findings add to the growing evidence that the mechanical behavior of the TMJ disc is highly influenced by loading direction and regional tissue properties. Overall, the results suggest that anisotropy and fiber orientation are key principles organizing joint and connective tissue biomechanics, resulting from the interaction between collagen fiber structure, extracellular matrix composition, and the strength and direction of applied loads.

## Data Availability

The raw data supporting the conclusions of this article will be made available by the authors, without undue reservation.

## References

[B1] Abraham A. C. Edwards C. R. Odegard G. M. Donahue T. L. H. (2011). Regional and fiber orientation dependent shear properties and anisotropy of bovine meniscus. J. Mech. Behav. Biomed. Mater. 4, 2024–2030. 10.1016/j.jmbbm.2011.06.022 22098902 PMC3222856

[B2] Angelo D. F. Morouço P. Alves N. Viana T. Santos F. González R. (2016). Choosing sheep (Ovis aries) as animal model for temporomandibular joint research: morphological, histological and biomechanical characterization of the joint disc. Morphologie 100, 223–233. 10.1016/j.morpho.2016.06.002 27450042

[B3] Ben-Zvi Y. Reznikov N. Shahar R. Weiner S. (2017). 3D architecture of trabecular bone in the pig mandible and femur: Inter-trabecular angle distributions. Front. Mater. 4, 29. 10.3389/fmats.2017.00029

[B4] Benavides E. Bilgen M. Al-Hafez B. Alrefae T. Wang Y. Spencer P. (2009). High-resolution magnetic resonance imaging and diffusion tensor imaging of the porcine temporomandibular joint disc. Dentomaxillofacial Radiol. 38, 148–155. 10.1259/dmfr/19195745 19225085

[B5] Chirani R. A. Jacq J.-J. Meriot P. Roux C. (2004). Temporomandibular joint: a methodology of magnetic resonance imaging 3-D reconstruction. Oral Surg. Oral Med. Oral Pathol. Oral Radiol. Endodontol. 97, 756–761. 10.1016/j.tripleo.2004.02.073 15184860

[B6] Cibis M. Jarvis K. Markl M. Rose M. Rigsby C. Barker A. J. (2015). The effect of resolution on viscous dissipation measured with 4D flow MRI in patients with fontan circulation: evaluation using computational fluid dynamics. J. Biomechanics 48, 2984–2989. 10.1016/j.jbiomech.2015.07.039 26298492 PMC5096445

[B7] Costa A. L. F. Yasuda C. L. Appenzeller S. Lopes S. L. P. C. Cendes F. (2008). Comparison of conventional MRI and 3D reconstruction model for evaluation of temporomandibular joint. Surg. Radiol. Anat. 30, 663–667. 10.1007/s00276-008-0400-z 18704257

[B8] Cui S. Guo Y. Fu Y. Zhang T. Zhang J. Gan Y. (2025). Inflammation-related collagen fibril destruction contributes to temporomandibular joint disc displacement *via* NF-κB activation. Int. J. Oral Sci. 17, 35. 10.1038/s41368-025-00352-0 40246831 PMC12006360

[B9] De Meurechy N. Aktan M. K. Boeckmans B. Huys S. Verwilghen D. R. Braem A. (2022). Surface wear in a custom manufactured temporomandibular joint prosthesis. J. Biomed. Mater Res. 110, 1425–1438. 10.1002/jbm.b.35010 35088936 PMC9306732

[B10] De Riu G. Stimolo M. Meloni S. M. Soma D. Pisano M. Sembronio S. (2013). Arthrocentesis and temporomandibular joint disorders: clinical and radiological results of a prospective study. Int. J. Dent. 2013, 1–8. 10.1155/2013/790648 24319462 PMC3844254

[B11] Eder J. Tonar Z. Schmid-Schwap M. Bristela M. Skolka A. Traxler H. (2018). Regional collagen fiber network in the articular disc of the human temporomandibular joint: biochemical 3-Tesla quantitative magnetic resonance imaging compared to quantitative histologic analysis of fiber arrangement. J. Oral Facial Pain Headache 32, 266–276. 10.11607/ofph.1879 30036886

[B12] Guerin H. A. L. Elliott D. M. (2006). Degeneration affects the fiber reorientation of human annulus fibrosus under tensile load. J. Biomechanics 39, 1410–1418. 10.1016/j.jbiomech.2005.04.007 15950233

[B13] Gutman S. Kim D. Tarafder S. Velez S. Jeong J. Lee C. H. (2018). Regionally variant collagen alignment correlates with viscoelastic properties of the disc of the human temporomandibular joint. Archives Oral Biol. 86, 1–6. 10.1016/j.archoralbio.2017.11.002 29128675 PMC5745264

[B14] Halilaj E. Moore D. C. Laidlaw D. H. Got C. J. Weiss A.-P. C. Ladd A. L. (2014). The morphology of the thumb carpometacarpal joint does not differ between men and women, but changes with aging and early osteoarthritis. J. Biomechanics 47, 2709–2714. 10.1016/j.jbiomech.2014.05.005 24909332 PMC4130650

[B15] Hallman J. J. Yoganandan N. Pintar F. A. (2011). Technique for chestband contour shape-mapping in lateral impact. J. Biomechanics 44, 2328–2332. 10.1016/j.jbiomech.2011.05.029 21676399 PMC3148324

[B16] Herring S. W. (2003). TMJ anatomy and animal models. J. Musculoskelet. Neuronal Interact. 3 (4), 391–394. 15758330 PMC2821032

[B17] Jiang N. Chen H. Zhang J. Cao P. Wang P. Hou Y. (2023). Decellularized-disc based allograft and xenograft prosthesis for the long-term precise reconstruction of temporomandibular joint disc. Acta Biomater. 159, 173–187. 10.1016/j.actbio.2023.01.042 36708853

[B18] Labus K. M. Kuiper J. P. Rawlinson J. Puttlitz C. M. (2021). Mechanical characterization and viscoelastic model of the ovine temporomandibular joint disc in indentation, uniaxial tension, and biaxial tension. J. Mech. Behav. Biomed. Mater. 116, 104300. 10.1016/j.jmbbm.2020.104300 33454627

[B19] Lake S. P. Miller K. S. Elliott D. M. Soslowsky L. J. (2010). Tensile properties and fiber alignment of human supraspinatus tendon in the transverse direction demonstrate inhomogeneity, nonlinearity, and regional isotropy. J. Biomechanics 43, 727–732. 10.1016/j.jbiomech.2009.10.017 19900677 PMC2823853

[B20] Lee J. D. Becker J. I. Larkin L. M. Almarza A. J. Kapila S. D. (2022). Morphologic and histologic characterization of sheep and porcine TMJ as large animal models for tissue engineering applications. Clin. Oral Invest 26, 5019–5027. 10.1007/s00784-022-04472-3 35359187 PMC9276584

[B21] Legemate K. Tarafder S. Jun Y. Lee C. H. (2016). Engineering human TMJ discs with protein-releasing 3D-Printed scaffolds. J. Dent. Res. 95, 800–807. 10.1177/0022034516642404 27053116

[B22] MacBarb R. F. Chen A. L. Hu J. C. Athanasiou K. A. (2013). Engineering functional anisotropy in fibrocartilage neotissues. Biomaterials 34, 9980–9989. 10.1016/j.biomaterials.2013.09.026 24075479 PMC3885350

[B23] Mirone A. Brun E. Gouillart E. Tafforeau P. Kieffer J. (2014). The PyHST2 hybrid distributed code for high speed tomographic reconstruction with iterative reconstruction and *a priori* knowledge capabilities. Nucl. Instrum. Methods Phys. Res. Sect. B Beam Interact. Mater. Atoms 324, 41–48. 10.1016/j.nimb.2013.09.030

[B24] Mölich J. Anuth S. Suuronen J.-P. Bortel E. Gerber J. Mattern E. (2025). Individual component-based parameter-adaptive segmentation approach for improved segmentation of synchrotron µCT data of osteocyte lacunae in bone tissue. Tomogr. Mater. Struct. 8, 100066. 10.1016/j.tmater.2025.100066

[B25] Nixdorf D. R. Greiser A. Hayes C. Gaalaas L. Groenke B. R. Fuglsig J. M. D. C. E. S. (2025). Comparison of a 0.55 T dental-dedicated magnetic resonance imaging system with a 1.5 T system in evaluation of the temporomandibular joint regarding subjective image quality assessment and rater agreement. Oral Surg. Oral Med. Oral Pathol. Oral Radiol. 140, 113–124. 10.1016/j.oooo.2025.02.011 40169337

[B26] Paganin D. Mayo S. C. Gureyev T. E. Miller P. R. Wilkins S. W. (2002). Simultaneous phase and amplitude extraction from a single defocused image of a homogeneous object. J. Microsc. 206, 33–40. 10.1046/j.1365-2818.2002.01010.x 12000561

[B27] Patil S. Bindra K. (2012). Morphology of the temporomandibular joint (TMJ) of sheep (*Ovis aries*). OJVM 2, 242–244. 10.4236/ojvm.2012.24039

[B28] Reed D. A. Yotsuya M. Gubareva P. Toth P. T. Bertagna A. (2019). Two-photon fluorescence and second harmonic generation characterization of extracellular matrix remodeling in post-injury murine temporomandibular joint osteoarthritis. PLoS ONE 14, e0214072. 10.1371/journal.pone.0214072 30897138 PMC6428409

[B29] Reznikov N. Liang H. McKee M. D. Piché N. (2022). Technical note: mapping of trabecular bone anisotropy and volume fraction in 3D using μCT images of the human calcaneus. Am. J. Biol. Anthropol. 177, 566–580. 10.1002/ajpa.24474

[B30] Rupp J. D. Flannagan C. A. C. Kuppa S. M. (2010). An injury risk curve for the hip for use in frontal impact crash testing. J. Biomechanics 43, 527–531. 10.1016/j.jbiomech.2009.09.038 19875117

[B31] Savignat M. Demondion X. Colard T. (2025). Thickness measurements and micro‐ CT imaging of human temporo‐mandibular discs. J. Anat. 247, 304–313. 10.1111/joa.14237 40045168 PMC12265026

[B32] She Y. Sun Y. Jiang N. (2024). The mechanics of tissue-engineered temporomandibular joint discs: current status and prospects for enhancement. J. Biomater. Appl. 39, 269–287. 10.1177/08853282241265059 39023922

[B33] Singh A. K. Khanal N. Chaulagain R. (2022). Advances in tissue engineering of the temporomandibular joint disc: an overview of current status and future directions. Int. J. Dent. 2022, 9696378. 10.1155/2022/9696378 35910087 PMC9337926

[B34] Socorro M. Dong X. Trbojevic S. Chung W. Brown B. N. Almarza A. (2025). The goat as a model for temporomandibular joint disc replacement: techniques for scaffold fixation. Br. J. Oral Maxillofac. Surg. 63, 91–97. 10.1016/j.bjoms.2024.10.233 39741089

[B35] Tanaka E. Van Eijden T. (2003). Biomechanical behavior of the temporomandibular joint disc. Crit. Rev. Oral Biol. Med. 14, 138–150. 10.1177/154411130301400207 12764076

[B36] Thomopoulos S. Marquez J. P. Weinberger B. Birman V. Genin G. M. (2006). Collagen fiber orientation at the tendon to bone insertion and its influence on stress concentrations. J. Biomechanics 39, 1842–1851. 10.1016/j.jbiomech.2005.05.021 16024026

[B37] Turunen M. J. Khayyeri H. Guizar-Sicairos M. Isaksson H. (2017). Effects of tissue fixation and dehydration on tendon collagen nanostructure. J. Struct. Biol. 199, 209–215. 10.1016/j.jsb.2017.07.009 28760694

[B38] Vogl T. J. Günther D. Weigl P. Scholtz J.-E. (2021). Diagnostic value of dynamic magnetic resonance imaging of temporomandibular joint dysfunction. Eur. J. Radiology Open 8, 100390. 10.1016/j.ejro.2021.100390 34926727 PMC8648939

[B39] Weitkamp T. Scheel M. Perrin J. Daniel G. King A. Le Roux V. (2022). Microtomography on the ANATOMIX beamline at synchrotron SOLEIL. J. Phys. Conf. Ser. 2380, 012122. 10.1088/1742-6596/2380/1/012122

