# Supplementary material for: 3D imaging and anisotropy mapping of the lamb disc for biomechanical and regenerative insights
Source: Front Physiol. 2026 Mar 5;17:1786152. doi: 10.3389/fphys.2026.1786152 (PMC12999453; doi:10.3389/fphys.2026.1786152)
Supplement: Supplementary file 2 [file DataSheet1.docx]

Supplementary Material

# Histological and virtual slices:

Comparison between histological and virtual slices of TMJ tissues demonstrated the effects of histological preparation. The fibrocartilage of the disc can shrink, and the slicing can cause dislocation of the tissue (Supplementary Figure 1A). Furthermore, the fiber bundles of the disc can show a wavier structure.

Although phase-contrast imaging mainly enhances edge and interface visibility, the reconstructed 3D data still depend on tissue density. This is because a residual attenuation signal contributes to image contrast. As a result, the histogram shows distinct peaks for different tissue types (Supplementary Figures 1B and 1C). The intensity relationship between hard and soft tissues, along with their spatial proximity, can affect the visibility of fine details in less-dense areas. Therefore, contrast adjustments are often necessary to improve visualization of both hard and soft tissues. Additionally, tissue segmentation allows for a more detailed analysis of their internal structures.


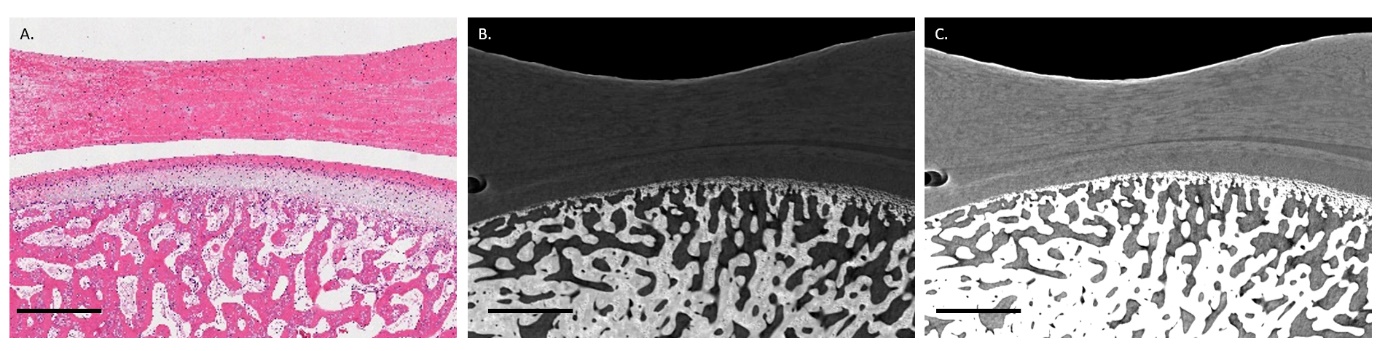


Supplementary Figure 1. Comparative histological and virtual slices characterizing the TMJ articular tissues: (A) Histological section (H&E stain) showing bone, articular cartilage, and TMJ disc. (B) Virtual slice in a corresponding area with contrast focusing on the bone structure. (C) Same area with contrast focusing on the disc/cartilage tissue. Scale bar = 2 mm.

# The Disc surface from different angles

The TMJ disc volume was systematically analyzed from multiple angles (Supplementary Figure 2). First, the entire disc volume was rendered to assess its overall shape and to locate regions with different fiber densities. The segmented fiber content was then overlaid to check the accuracy of segmentation (Supplementary Figure 2, left column). Next, the eigenvector fields were examined to evaluate fiber orientation in various regions (Supplementary Figure 2, middle column). Lastly, vector anisotropy was visualized to gain a better understanding of the disc’s structural organization (Supplementary Figure 2, right column).


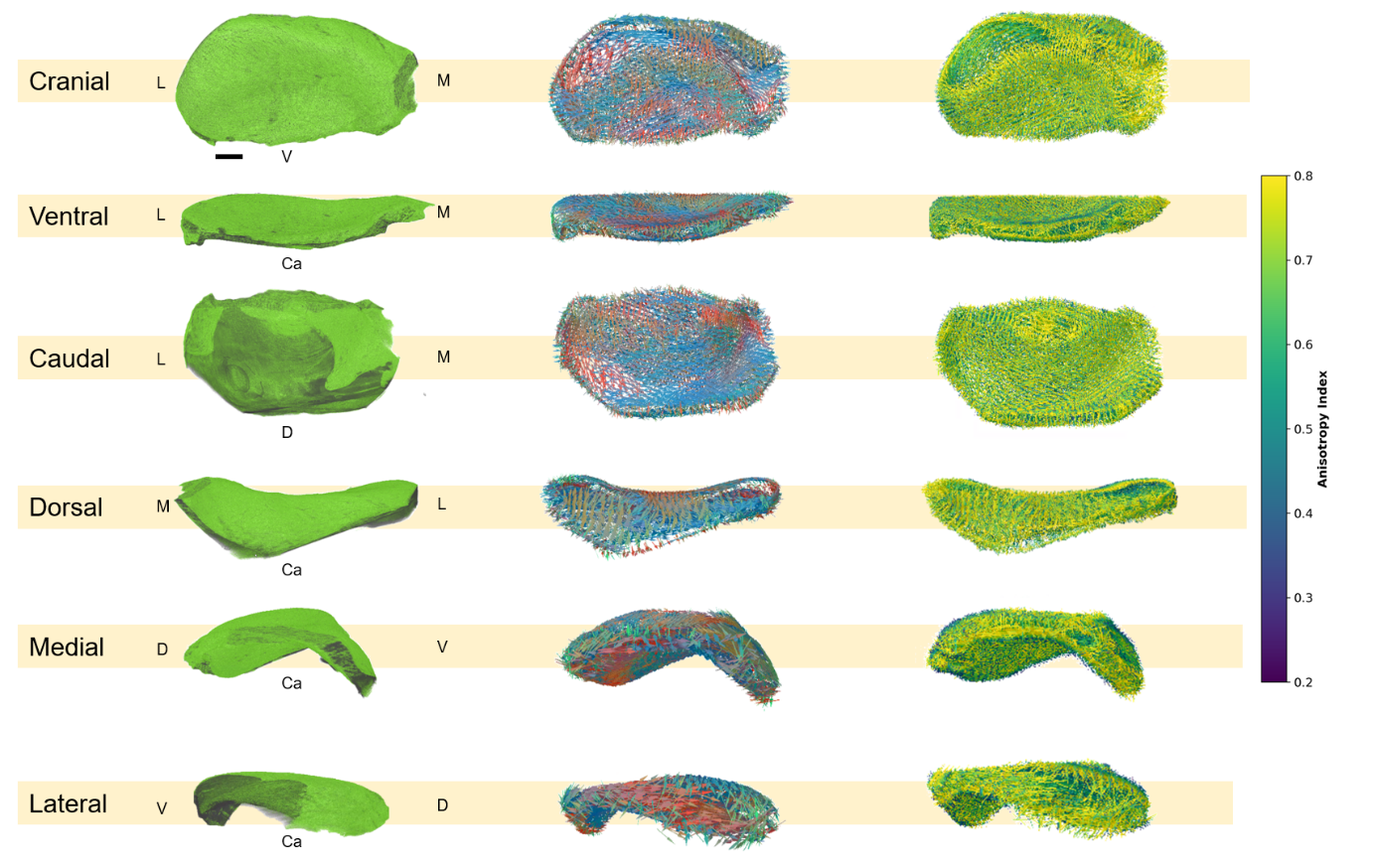


Supplementary Figure 2. Regional 3D morphology and surface fiber orientation of the articular disc viewed from all anatomical directions: Surface renderings of the articular disc (left column) are paired with corresponding fiber orientation vector maps (middle column), and anisotropy maps for each anatomical perspective: cranial, ventral, caudal, dorsal, medial, and lateral views. Directional axes are labeled on each panel (L = lateral, M = medial, V = ventral, D = dorsal, Cr = cranial, Ca = caudal). Fiber vectors are color-coded based on their dominant anatomical axis component: blue = lateral–medial (X-axis), red = ventral–dorsal (Y-axis), and green = cranial–caudal (Z-axis). Intermediate vectors have blended colors. Anisotropy index colors indicate anisotropy index (0.2 = blue, low order; 0.8 = yellow, high order). Scale bar = 2 mm.

# Fiber Orientation and Anisotropy Analysis of the TMJ Disc

Before conducting an in-depth analysis of the anisotropy of the TMJ disc’s fibers in different planes, all the fibers were counted, and their mean and median anisotropy values were calculated (Supplementary Figure 3A). Additionally, a simple histogram visualization of all fibers in various planes was examined (Supplementary Figure 3B-D), which highlighted the need for a more two-dimensional depiction of the fiber distribution within the sagittal, frontal, and transverse planes (Figure 4, Manuscript).


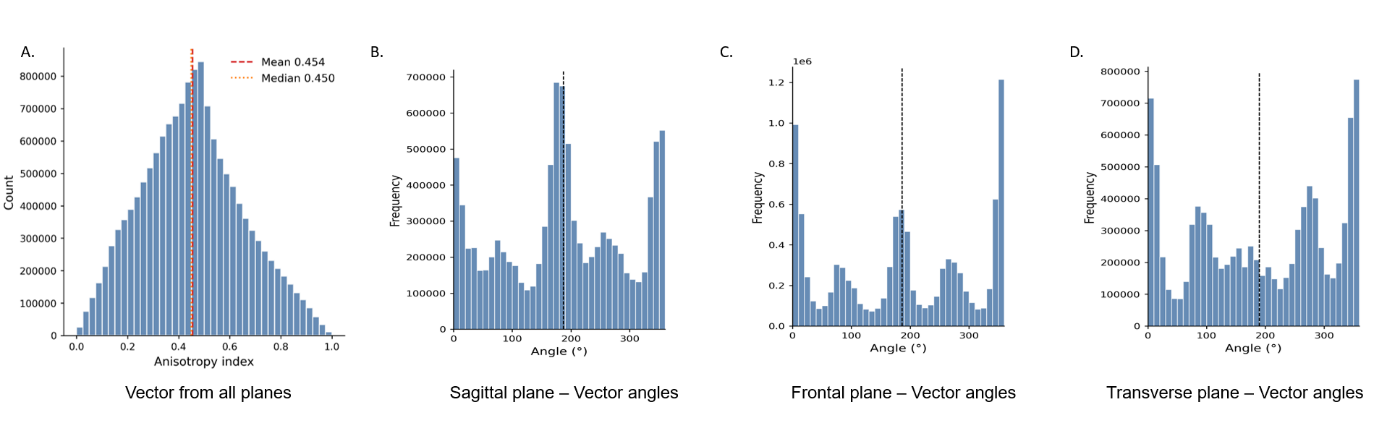


Supplementary Figure 3. Distribution of anisotropy index and fiber orientation frequency across the lamb TMJ disc: (A) Histogram of the anisotropy index for all sampled voxels showing a near-normal distribution centered around a mean value of 0.45, indicating moderate global anisotropy across the disc. (B–D) Vector angle distributions frequency for the sagittal (B), frontal (C), and transverse (D) planes. All planes display bimodal peaks near 0° and 180°, corresponding to fibers oriented predominantly along the craniocaudal (sagittal) and lateromedial (frontal and transverse) axes. Intermediate angle ranges exhibit lower frequencies.
